# Novel Insights into Epigenetic Regulation of IL6 Pathway: In Silico Perspective on Inflammation and Cancer Relationship

**DOI:** 10.3390/ijms221810172

**Published:** 2021-09-21

**Authors:** Saverio Candido, Barbara Maria Rita Tomasello, Alessandro Lavoro, Luca Falzone, Giuseppe Gattuso, Massimo Libra

**Affiliations:** 1 Department of Biomedical and Biotechnological Sciences, University of Catania, 95123 Catania, Italy; alessandrolavoro@ymail.com (A.L.); peppeg9305@gmail.com (G.G.); mlibra@unict.it (M.L.); 2 Research Center for Prevention, Diagnosis and Treatment of Cancer, University of Catania, 95123 Catania, Italy; 3 Drug Sciences, University of Catania, 95124 Catania, Italy; 4 Epidemiology Unit, IRCCS Istituto Nazionale Tumori “Fondazione G. Pascale”, 80131 Naples, Italy; l.falzone@istitutotumori.na.it

**Keywords:** IL6, IL6R, IL6ST, tumor, bioinformatics, epigenetics, TCGA, methylation, miRNAs, therapeutic approaches

## Abstract

IL-6 pathway is abnormally hyperactivated in several cancers triggering tumor cell growth and immune system inhibition. Along with genomic mutation, the IL6 pathway gene expression can be affected by DNA methylation, microRNAs, and post-translational modifications. Computational analysis was performed on the Cancer Genome Atlas (TCGA) datasets to explore the role of IL6, IL6R, IL6ST, and IL6R transmembrane isoform expression and their epigenetic regulation in different cancer types. IL6 was significantly modulated in 70% of tumor types, revealing either up- or down-regulation in an approximately equal number of tumors. Furthermore, IL6R and IL6ST were downregulated in more than 10 tumors. Interestingly, the correlation analysis demonstrated that only the IL6R expression was negatively affected by the DNA methylation within the promoter region in most tumors. Meanwhile, only the IL6ST expression was extensively modulated by miRNAs including miR-182-5p, which also directly targeted all three genes. In addition, IL6 upregulated miR-181a-3p, mirR-214-3p, miR-18a-5p, and miR-938, which in turn inhibited the expression of IL6 receptors. Finally, the patients’ survival rate was significantly affected by analyzed targets in some tumors. Our results suggest the relevance of epigenetic regulation of IL6 signaling and pave the way for further studies to validate these findings and to assess the prognostic and therapeutic predictive value of these epigenetic markers on the clinical outcome and survival of cancer patients.

## 1. Introduction

The vast majority of clinical and experimental evidences suggest that acute inflammation could promote tumor eradication by exerting an immunoprotective effect [1]. Conversely, tumor growth and invasion could be facilitated by chronic immune stimulation [1,2]. However, all these responses are orchestrated by soluble mediators (e.g., chemokines and cytokines) including tumor necrosis factor alpha (TNF-α) and Interleukin-6 (IL-6) secreted by either host immune cells or tumor cells themselves [2,3,4]. Although the pleiotropic cytokine IL6 is involved in multiple physiological functions, it is also relevant to inflammatory diseases and cancer [5]. IL6 exerts its effects by engaging its receptor IL6R along with two subunits of gp130 (glycoprotein 130), also known as IL6ST (Interleukin 6 Signal Transducer), to form a hexameric complex which in turn activates two different signaling pathways [6]. Firstly, a classical pathway through its transmembrane receptor IL-6R (cis-signaling pathway). Secondly, trans-signaling pathway through a soluble IL-6-receptor-α (sIL-6R) which binds IL6 forming a complex able to interact and activate any cell expressing the receptor subunit glycoprotein gp130 [6,7]. Importantly, the IL6 trans-signaling pathway is involved in most of the harmful effects of IL-6 in chronic inflammatory diseases and in cancer where it leads to switch on IL6-signaling in tumor or stromal cells expressing zero or low level of IL6R [7].

In the tumor microenvironment, the IL-6 signaling pathway activation results in a dual effect [8,9]. The first one is the stimulation of cancer cell proliferation, survival, invasiveness and metastasis and the cancer stem cells self-renew [10]. The second effect is on immune system, which is strongly suppressed. Moreover, IL-6 stimulates the production of pro-inflammatory and pro-angiogenic factors, including Interleukin-1 beta (IL-1β), Interleukin-8 (IL-8), Granulocyte-Macrophage Colony-Stimulating Factor (GM-CSF), and Vascular Endothelial Growth Factor (VEGF), which affect immune and non-immune cells of the tumor microenvironment in an autocrine and/or paracrine fashion [11]. The abnormal hyperactivation of IL6 pathway is usually associated with a poor clinical prognosis in many types of cancer [12,13,14]. The extent of the damaging effect following the IL-6 signaling pathway hyperactivation may be related to the changes in the expression of its nodes due to the dysregulation of some transcription factors (TF) and/or genomic alteration of TF consensus binding sequence within the promoter region [15,16,17]. Furthermore, a crucial role is played by the amount of sIL-6R produced by proteolytic cleavage of the IL-6R by ADAM17 or ADAM10 proteases or by alternative splicing, which generates distinct IL6R isoforms including those lacking transmembrane domain [18,19].

Epigenetic changes can modify the normal expression pattern of genes in some diseases including cancer [20]. Indeed, it is well known that aberrant epigenetic modifications are frequent in cancer and may dysregulate genes involved in tumorigenesis including various inflammatory cytokines [21,22]. Indeed, DNA methylation plays a crucial role in gene expression affecting the binding of transcription factors and enhancer elements in their specific consensus sequences [23]. Furthermore, it was demonstrated that intragenic DNA methylation modulated the alternative splicing of several genes [24,25,26]. Regarding the epigenetic modulation of IL6 pathway, the IL-6 expression is affected by DNA methylation of promoter region in several cancers [27,28]. Conversely, it was demonstrated that IL-6 stimulation altered the DNA methylation patterns of related genes promoting tumorigenesis [29,30]. However, the DNA methylation patterns of IL6R and IL6ST receptors has been barely investigated in cancer.

Along with DNA methylation, another epigenetic mechanism implicated in the regulation of gene expression is the transcriptional silencing mediated by the microRNAs (miRNAs) [31]. In particular, the miRNAs are short small non-coding RNA molecules involved in mRNA degradation of target genes and/or in the mRNA translation into protein by binding specific sequences within untranslated region (UTR) and coding sequence (CDS), respectively [31,32]. Over the past two decades, the involvement of miRNAs expression in the development of cancer is the most studied epigenetic mechanism [33,34,35,36]. miRNAs play a dual role in cancer by acting either as oncogenes or tumor-suppressor depending on the tumoral context [37]. Several studies reported that miRNAs affect cancer-related inflammatory pathways in oral squamous cell carcinoma, chronic lymphocytic leukemia, and esophageal cancer [38,39,40]. A potential link between miRNAs and inflammation was also proposed for cholangiocarcinoma by Lin et al. (2016) that suggested the downregulation of let-7c, miR-99a and miR-125b promoted tumor pathogenesis and progression by targeting IL-6, IL-6R and Insulin-like Growth Factor 1 (IGF1) [41].

Recently, Yokomizo et al. (2019), using different methods including in vitro, in vivo, and clinical approaches demonstrated that the reduction of tumor-suppressor miR-34a expression is strongly correlated with the pathogenesis of epithelial ovarian cancer inducing the upregulation of IL-6R and the consequent dysregulation of IL-6/STAT3 target genes [42].

Since the onset of inflammation and carcinogenesis can be regulated by epigenetic events, we aimed to investigate the interplay among DNA methylation, miRNA expression, and gene expression of IL6, IL6R, including transmembrane isoform, and IL6ST in different tumor types.

To this purpose, integrative analysis of cancer genomic and epigenomic data from TCGA datasets was performed to provide new insights on epigenetic regulations of IL6, IL6R, and IL6ST in cancer. Furthermore, this computational approach could lead to identify new diagnostic and prognostic epigenetic markers as well as specific therapeutic targets for the cancer treatment

## 2. Results

### 2.1. Gene Expression and Survival Analysis

Differential analysis of IL6, IL6R, and IL6ST revealed that these genes were significantly modulated in most of the selected TCGA tumor cohorts (n = 33) compared to normal tissues (Table 1). In particular, IL6 gene was modulated in 70% of tumor types (23 of 33) showing an up-regulation in 13 tumors and a down-regulation in 10. Of note, the highest increment of IL6 was observed for the large B-cell lymphoma (FC: 6.39) and testicular cancer (FC: 5.19) whereas IL6 was strongly downregulated in ocular melanomas (FC: −11.90) and breast (FC: −7.37) cancer (Table 1). Furthermore, IL6R expression was down-regulated in 20 tumors and weakly upregulated in 5 tumors. In particular, the intestinal tumors (colon and rectal cancers), sarcoma, thymoma, uterine carcinosarcoma, and large B-cell lymphoma showed a strong IL6R down-regulation (FC ≤ −5). Regarding IL6ST gene, it was moderately downregulated in 12 tumors while it was only overexpressed in 4 tumors. In addition, both IL6R and IL6ST were simultaneously down-regulated in nearly 25% of TCGA tumors (Table 1). Notably, only seven tumors (21%) showed concomitant modulation of IL6, IL6R, and IL6ST. Among these, only kidney clear cell carcinoma displayed the upregulation of all three genes while they were downregulated in lung squamous cell carcinoma. Furthermore, bladder, cervical, endometrioid, and uterine cancers, displayed IL6 upregulation and both IL6R and IL6ST downregulation. Finally, IL6 and IL6R genes were both upregulated in glioblastoma, head and neck, kidney clear cell carcinoma, and testicular cancer. Conversely, they were both downregulated in acute myeloid leukemia, breast, kidney chromophobe, liver, ocular and thyroid cancers, lung squamous cell carcinoma, and mesothelioma.

The overall survival analysis was performed stratifying the cancer samples of each TCGA cohort based on the expression mean value of IL6, IL6R, and IL6ST by using UCSC Xena tool. The expression levels of each gene in the different tumor types are reported in Figure S1.

The overall survival analysis revealed that IL6 expression was predictive of poor prognosis in eight tumors including kidney clear cell carcinoma (log rank value: 31.46) and lower grade glioma (log rank value: 12.38) that showed the highest log-rank values. On the other hand, IL6 expression represented a favorable prognostic indicator only in sarcoma patients (Table 1). Moreover, IL6R predictive value for cancer patients’ overall survival was significant (*p* ≤ 0.05) in six tumor types, however displaying a different trend among these. Furthermore, IL6ST was positively related to survival in head and neck cancer, kidney clear cell carcinoma, and lung adenocarcinoma.

Regarding the Progression Free Interval (PFI) analysis, IL6 was mainly associated with unfavorable survival, whereas the IL6R and IL6ST showed a variable trend across tumors. Interestingly, all three genes were significantly associated with OS and PFI in kidney clear cell carcinoma in which IL6 had a negative prognostic significance while IL6R and IL6ST were associated with a favorable prognosis (Table 1).

### 2.2. IL6R Exon Expression and Survival Analysis

To evaluate the relative abundance of IL6R transmembrane isoform, the IL6R Exon 9 and Exon 2 expression data were retrieved for all TCGA tumor types (Figure S2).

The differential analysis revealed that 58% of tumors showed a moderate significant increase in relative expression of Exon 9-retaining IL6R isoform (FC ≥ 1.4—*p* ≤ 0.05) especially for the acute myeloid leukemia displayed a FC equal to 2.53 (Table S1). The TCGA SpliceSeq tool analysis demonstrated that the percent-spliced-in (PSI) was higher than 0.9 for all TCGA tumors, indicating that Exon 9 skipping was a sporadic event (Figure S3).

OS and PFI analyses were performed stratifying the tumor samples according to the relative expression of IL6R Exon 9 calculated for each sample as difference between log2 (Exon 9) and log2 Exon 2. The results indicated that OS was positively affected only in lung adenocarcinoma (Log-rank: 3.97—*p* ≤ 0.05). Furthermore, PFI was reduced in lower grade glioma patients with Exon 9 relative expression above mean value (Log-rank: 4.31—*p* ≤ 0.05) while the Exon 9 relative expression was associated with a favorable prognosis in ocular melanoma (Log-rank: 3.92—*p* ≤ 0.05) (Table S1).

### 2.3. DNA Methylation and Survival Analysis

**Figure 1:**
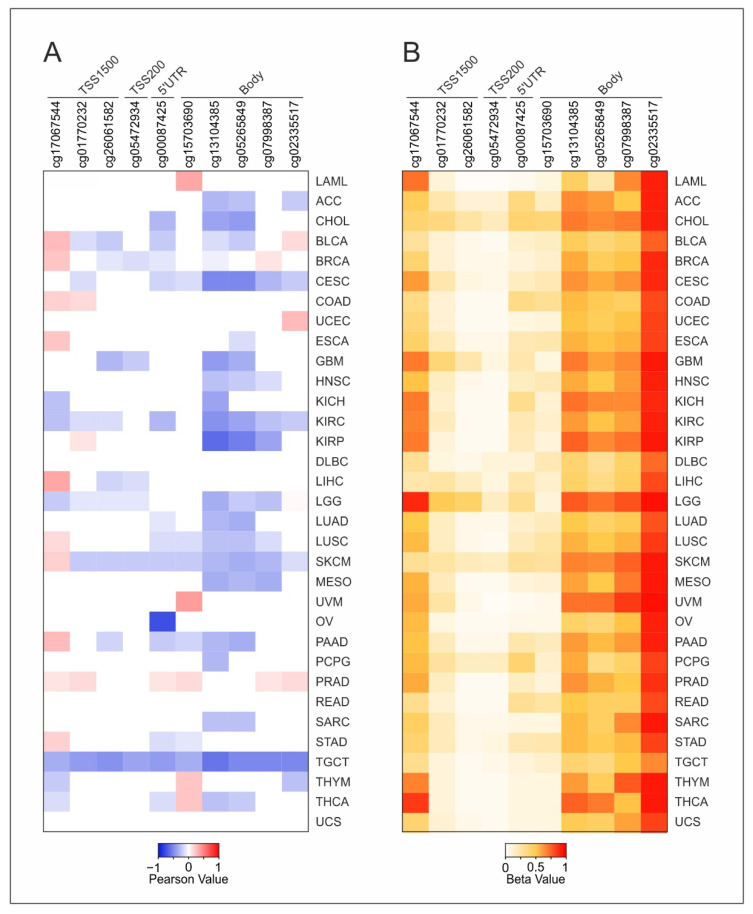
Correlation analysis between IL6 expression and DNA methylation for each tumor type. (**A**) The heatmap shows the significant (*p* ≤ 0.05) Pearson’s correlation coefficients ≤ −0.3 (blue) or ≥ 0.3 (red). (**B**) DNA methylation levels of IL6 CG probesets for each tumor type. The heatmap cell represents the mean values of methylation levels for each CG probeset.

**Figure 2:**
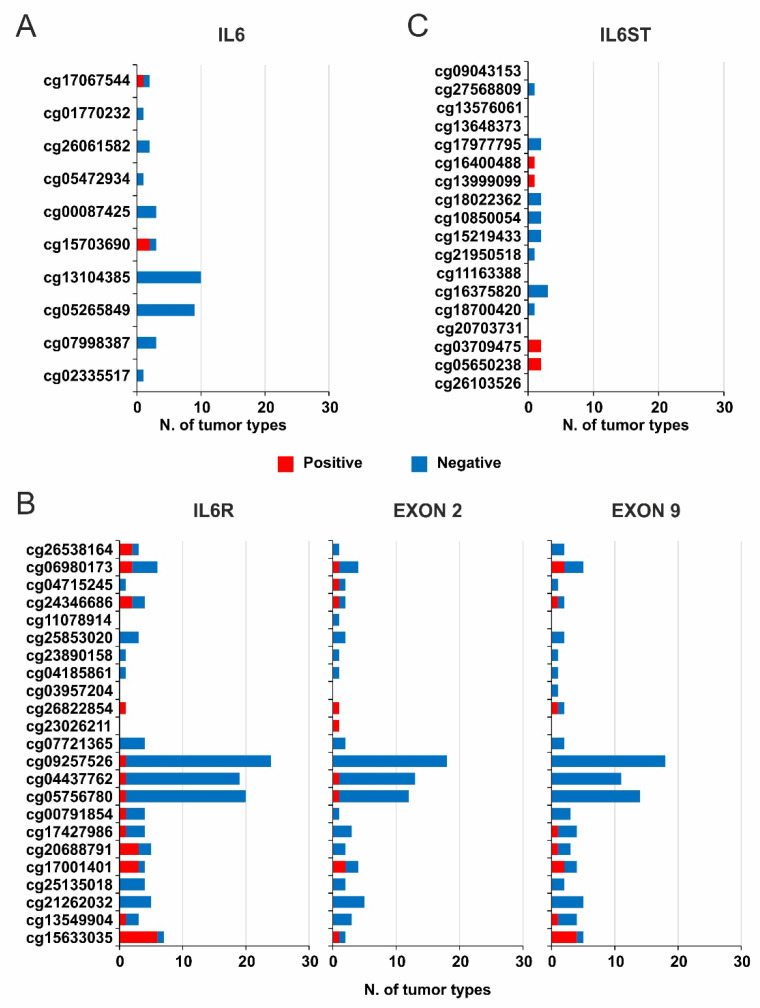
Overview of correlation analysis between IL6, IL6R, and IL6ST gene expression and CG probesets methylation. The number of CG probesets of IL6 (**A**), IL6R, IL6R Exon 2 and 9 (**B**), and IL6ST (**C**) showing Pearson’s r ≤ −0.3 or ≥ 0.3 (*p* ≤ 0.05) was evaluated in all TCGA tumor types.

The impact of DNA Methylation on the IL6, IL6R, and IL6ST gene expression was evaluated by the correlation analysis with the methylation levels of CG probesets within each gene. The correlation analysis revealed that IL6 expression was mainly negatively correlated with the probesets methylation in most tumor types (Figure 1 and Table S2). In particular, the cg13104385 and cg05265849 probesets were always negatively correlated (r ≤ −0.3) with IL6 expression in 10 and 9 tumors, respectively. Conversely, a positive correlation was observed in liver cancer for cg17067544 while the cg15703690 was positively correlated in ocular melanoma and acute myeloid leukemia (Figure 1A and Figure 2A and Table S2).

Interestingly, the CG probesets from cg13104385 to cg02335517 were widely hyper-methylated in all tumors. On the other hand, the promoter region CG probesets (from TSS1500 to 5′UTR) were hypomethylated except for the first cg17067544 probeset within the TSS1500 region, which was widely hypermethylated in all tumors (Figure 1B).

**Figure 3:**
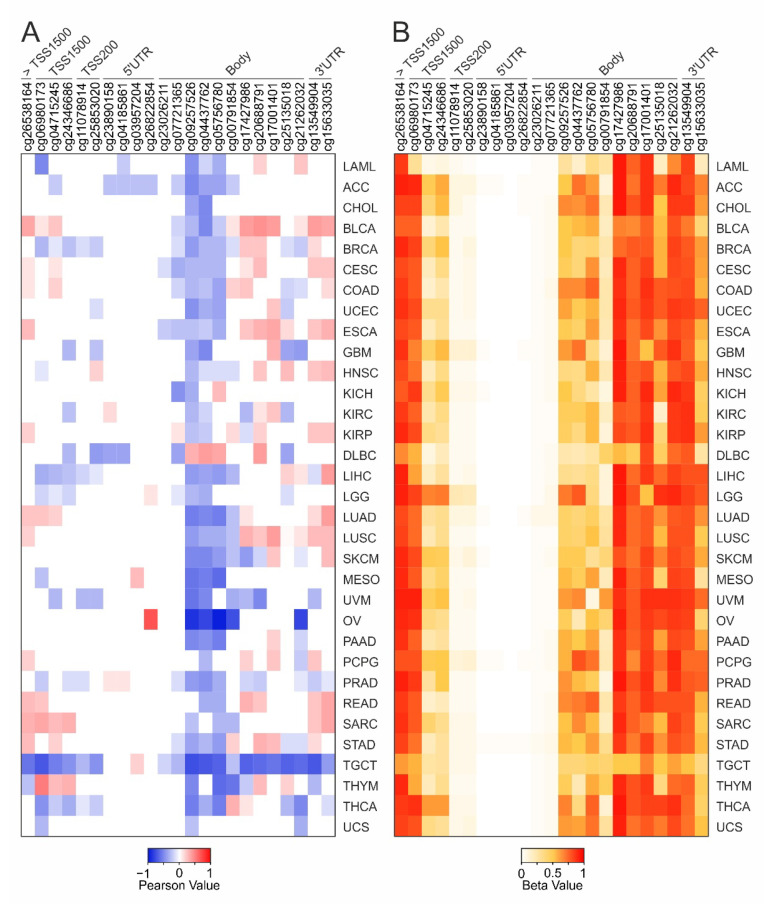
Correlation analysis between IL6R expression and DNA methylation for each tumor type. (**A**) The heatmap shows the significant (*p* ≤ 0.05) Pearson’s correlation coefficients ≤ −0.3 (blue) or ≥ 0.3 (red). (**B**) DNA methylation levels of IL6R CG probesets for each tumor type. The heatmap cell represents the mean values of methylation levels for each CG probeset.

When the correlation analysis was conducted between IL6R expression and DNA methylation, the cg09257526, cg04437762, and cg05756780 probesets within body region were negatively correlated (r ≤ −0.3) in more than 50% of tumors. Interestingly, a positive correlation was only observed in large B-cell lymphoma (Figure 2B and Figure 3A and Table S3).

Conversely, a large number of CG probesets included in the 3′-end of gene body and 3′UTR regions were generally positively correlated. It is remarkable that the aforementioned cg09257526, cg04437762, and cg05756780 probesets were also moderately hypermethylated in most of tumor types (Figure 3B). Besides, a strong hypermethylation was observed in some CG probesets within the TSS1500 region (cg26538164 and cg06980173) and the 3′-end of IL6R gene (from cg17427986 to cg15633035). Finally, the TSS200 and 5′UTR regions were uniformly hypomethylated in all evaluated tumors (Figure 3B).

To evaluate the role of DNA methylation in the regulation of alternative splicing of IL6R Exon 9, the correlation analysis was conducted between either IL6R Exon 9 or Exon 2 expression and methylation levels of each CG probeset of IL6R. This analysis showed few differences between the Exon 9 and 2 (Figure 2B, Figure S4 bold square). Besides, these results were comparable to those obtained when total IL6R gene expression was correlated to CG probesets methylation (Figure 2B and Figure 3A).

**Figure 4:**
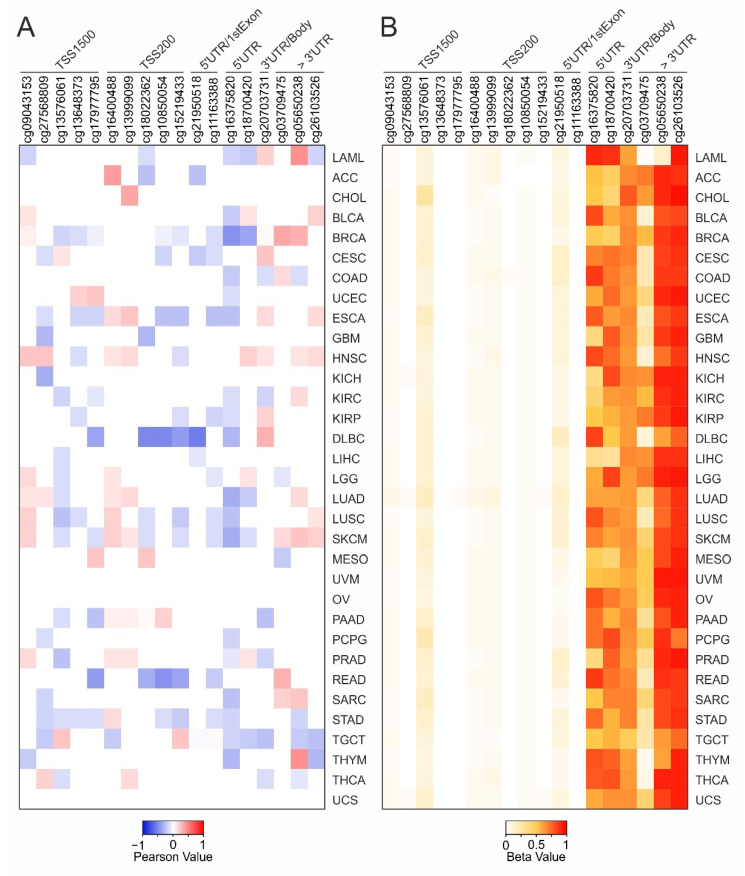
Correlation analysis between IL6ST expression and DNA methylation for each tumor type. (**A**) The heatmap shows the significant (*p* ≤ 0.05) Pearson’s correlation coefficients ≤ −0.3 (blue) or ≥ 0.3 (red). (**B**) DNA methylation levels of IL6ST CG probesets for each tumor type. The heatmap cell represents the mean values of methylation levels for each CG probeset.

Finally, inconclusive results were obtained when IL6ST gene expression was correlated to DNA methylation for each TCGA tumor (Figure 4A, Table S4). Indeed, the 66.7% of IL6ST CG probesets were significantly correlated (−0.3 ≤ Pearson’s R ≥ 0.3; *p* ≤ 0.05) in less than four tumor types (Figure 2C). Notably, moderate/high methylation levels were observed for CG probesets within the body and 3′UTR regions of IL6ST gene (Figure 4B).

To understand the clinical significance of DNA methylation of IL6, IL6R, and IL6ST genes, the OS and PFI analyses were performed stratifying the TCGA tumor samples according to the methylation levels of the highly representative cg13104385 and cg05265849 probesets of IL6 (negatively correlated in more than five tumor types) and cg09257526, cg04437762, and cg05756780 relative to IL6R (negatively correlated in more than 10 tumor types) (Table 2).

Interestingly, the methylation of cg13104385 and cg05265849 probesets were generally associated to a favorable prognosis except for the melanoma in which the methylation of cg13104385 probeset represented a negative prognostic factor (log rank: 5.75). Conversely, the methylation levels of selected IL6R CG probesets mostly negatively affected the patient survival. However, the methylation status of some IL6R CG probesets represented a favorable prognostic factor in breast cancer, glioblastoma, lower grade glioma, melanoma, and thymoma (Table 2).

### 2.4. miRNAs Expression and Survival Analysis

The role of miRNAs in the modulation of IL6, IL6R, and IL6ST gene expression was assessed by performing correlation analysis between the expression of IL6, IL6R, and IL6ST genes (IlluminaHiSeq pancan normalized) and miRNAs expression data (IlluminaHiseq) available for all TCGA tumors (Tables S5–S7). Similarly, the correlation analysis was also carried out for Exon 2 and 9 (Tables S8 and S9).

**Figure 5:**
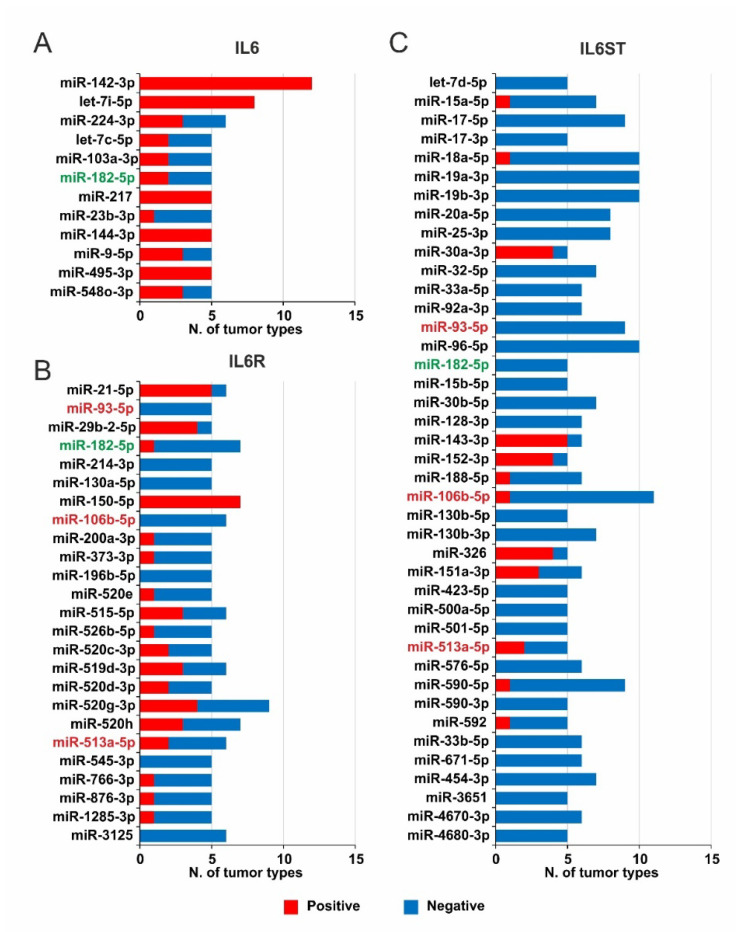
Overview of selected miRNAs correlated with the expression of IL6, IL6R, and IL6ST. The number of miRNAs targeting IL6 (**A**), IL6R (**B**), and IL6ST (**C**) (mirDIP class score: high/very high) was evaluated in all TCGA tumor types. The miRNAs with Pearson’s r ≤ −0.3 or ≥ 0.3 (*p* ≤ 0.05) in at least 5 different tumor types are shown. It is reported, in green font, the miRNAs shared among IL6, IL6R, and IL6ST, whereas in red font those correlated to both IL6R and IL6ST.

The results outlined that more than 60% of the total mirDIP-predicted miRNAs were significantly correlated (Pearson’s correlation value ≥ 0.3 or ≤ −0.3; *p* ≤ 0.05) with the expression of each gene in at least one tumor type (IL6: 67%, IL6R: 70% and IL6ST: 64%) (Tables S5–S7). The correlation pattern for Exon 2 and 9 was similar, a massive 72% of all miRNAs were significantly correlated with both IL6R Exon 2 and 9 expression levels (Tables S8 and S9). Furthermore, miRNAs showing both Pearson’s r ≥ 0.3 or ≤ −0.3 (*p* ≤ 0.05) and a high/very high mirDIP class score in at least 5 tumors were selected to reveal the most significant miRNAs that could downregulate the expression of IL6, IL6R, and IL6ST (Figure 5).

The application of these stringent criteria allowed us to highlight that 12 miRNAs were highly correlated with IL6, showing a widely positive correlation (Figure 5A). In particular, the results for miR-142-3p and let-7i-5p showed a totally positive correlation in more than 7 tumor types (Figure 5A, Table S5). Among these, kidney chromophobe, bladder, and liver cancers displayed positive correlation for both miR-142-3p and let-7i-5p miRNAs (Table S5). Conversely, 7 of 25 miRNAs highly associated with IL6R expression were always negatively correlated in at least 5 tumor types, while miR-150-5p was positively correlated in 7 tumors (Figure 5B). Remarkably, the correlation analysis for IL6ST allowed to select 41 relevant miRNAs according to the aforementioned criteria and 70.73% was always negatively correlated (Figure 5C). Interestingly, miR-182-5p significantly correlated with IL6, IL6R, and IL6ST genes while the miR-106b-5p, miR-513a-5p, and miR-93-5p correlated only with IL6R and IL6ST (Figure 5).

**Figure 6:**
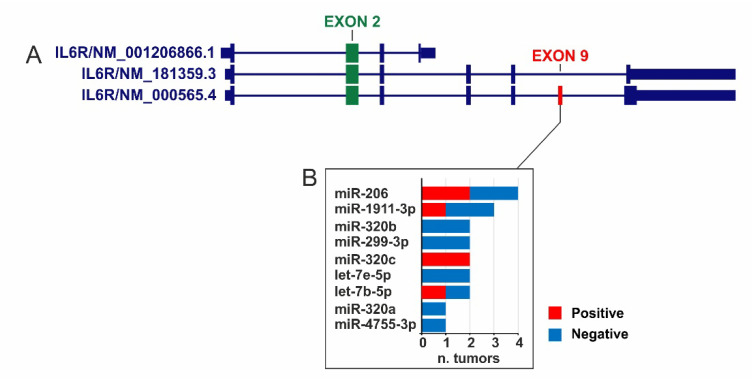
Correlation analysis of miRNAs targeting the sequence of Exon 9. (**A**) IL6R gene isoform according to RefSeq annotation (assembly: GRCh37/hg19). (**B**) In the left box miRNAs targeting Exon 9 with Pearson’s r ≤ −0.3 or ≥ 0.3 (*p* ≤ 0.05) are described. For each miRNA the number of TCGA tumors in which this is significantly correlated is reported.

In addition, STarMirDB analysis identified 9 miRNAs targeting IL6R Exon 9 (LogitProb ≥ 0.5) in at least 1 tumor type (Figure 6). These miRNAs were positively correlated in 6 tumors while 13 tumors showed a negative correlation (Figure 6B).

Notably, the miR-206, miR-320b, and let-7b-5p were completely negatively correlated with IL6R Exon 9 expression in large B-cell lymphoma. Similarly, the miR-320a and miR-320b showed negative correlation in uveal melanoma while the miR-206 and let-7e-5p were negatively correlate in testicular cancer (Table S9). Finally, no overlapping was observed between the selected miRNAs targeting IL6R Exon 9 (Figure 6B) and those targeting all the IL6R isoforms (Figure 5B).

**Figure 7:**
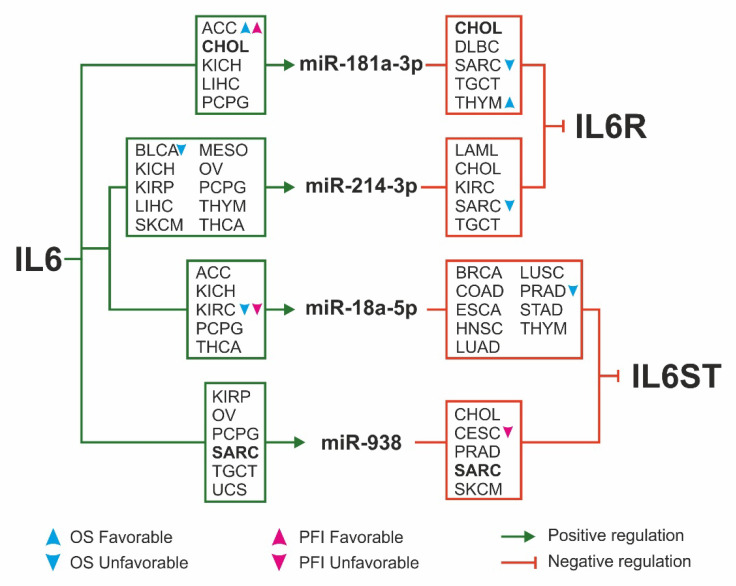
Negative feedback on IL6 receptors mediated by IL6-dependent miRNAs. The miRNAs positively correlated with IL6 and negatively correlated with IL6R and IL6ST in at least 5 different tumors are shown. In boxes are listed the tumor types showing miRNAs positively correlated with IL6 (green boxes) and negatively correlated with IL6R and IL6ST (red boxes). The tumors in which both correlations are satisfied are highlighted in bold. The prognostic significance of selected miRNAs for each tumor is indicated by the blue (OS) and magenta (PFI) arrowhead. Abbreviations: ACC: Adrenocortical Cancer; BLCA: Bladder Cancer; BRCA: Breast Cancer; CESC: Cervical Cancer; CHOL: Bile Duct Cancer; COAD: Colon Cancer; DLBC: Large B(-)cell Lymphoma; ESCA: Esophageal Cancer; HNSC: Head and Neck Cancer; KICH: Kidney Chromophobe; KIRC: Kidney Clear Cell Carcinoma; KIRP: Kidney Papillary Cell Carcinoma; LAML: Acute Myeloid Leukemia; LIHC: Liver Cancer; LUAD: Lung Adenocarcinoma; LUSC: Lung Squamous Cell Carcinoma; MESO: Mesothelioma; OV: Ovarian Cancer; PCPG: Pheochromocytoma and Paraganglioma; PRAD: Prostate Cancer; SARC: Sarcoma; SKCM: Melanoma; STAD: Stomach Cancer; TGCT: Testicular Cancer; THCA: Thyroid Cancer; THYM: Thymoma; UCS: Uterine Carcinosarcoma.

To understand the role of miRNAs in IL6 pathway regulation, the miRNAs positively correlated with IL6 (Pearson’s r ≥ 0.3, *p* ≤ 0.05) were overlapped with miRNAs negatively correlated with IL6R and IL6ST (Pearson’s r ≤ −0.3, *p* ≤ 0.05; mirDIP score ≥ medium). Only the miRNAs that fulfilled the aforementioned criteria in at least 5 tumors were included in the analysis. The results showed that miR-181a-3p, miR-214-3p, miR-18a-5p, and miR-938 were positively correlated with IL6. Among these, the first two miRNAs were also putative inhibitors of IL6R expression while miR-18a-5p and miR-938 were predicted to target IL6ST (Figure 7).

It is noteworthy that this correlation pattern was fulfilled for miR-181a-3p in bile duct cancer tumor and for miR-938 in sarcoma. Additionally, OS and PFI analysis were performed for the expression of miR-181a-3p, miR-214-3p, miR-18a-5p, and miR-938 (Table S10). Surprising, miR-18-a-5p was associated to an unfavorable prognosis in all these tumors listed as follows: endometrioid cancer, kidney clear cell carcinoma, liver cancer, melanoma, mesothelioma, prostate cancer, and sarcoma.

As shown in Figure 7, the survival was affected by these miRNAs in few tumors showing a tumor-type-dependent prognostic significance.

## 3. Discussion

The role of chronic inflammation in cancer development and progression has been widely investigated over the last decades. Several mechanisms, including immunity cells activation and the release of a plethora of proinflammatory mediator, are responsible for the growth and proliferation of cancer-initiating cells [43,44]. A growing number of studies have corroborated the role of the proinflammatory IL-6 in influencing the main aspects of cancer biology. An emerging research field deals with the understanding of the tumor cell response to IL-6 taking into account the precise regulation of IL-6 receptor complex consisting of IL6-R and gp130 proteins, which also exists in membrane-bound and/or soluble forms [6]. Notably, these receptors are modulated at different levels ranging from genetic regulation to post-translation modifications such as proteolytic cleavage mediated by ADAMs proteinases [18,19]. However, there appears to be dearth of studies exhaustively investigating the epigenetic modulations of IL-6 and its receptor complex in cancer.

Our bioinformatic analysis revealed that IL6, IL6R, and IL6ST genes were significantly modulated in most of the cancers showing a tumor-dependent pattern. For instance, among hematological malignancies we observed that IL6 was upregulated in large B-cell lymphoma while acute myeloid leukemia patients expressed lower levels of IL6 compared to healthy controls. These evidences agreed with the literature data which demonstrated that IL-6 is predominantly expressed in B-cell tumors with a key role in cell proliferation [45]. Similarly, heterogeneous expression profiling of IL-6 was obtained analyzing the epithelial-derived tumors, of which cervical and pancreatic cancers showed the highest IL6 expression levels while breast and lung cancers showed the lowest ones. These results were partially in conflict with the literature as IL-6 is associated with progression of pancreatic and lung cancers as well [46,47,48]. Finally, concordant results were achieved for ovarian and testicular cancers in which the rise of IL6 transcript levels corroborates the role of IL-6 in the physiopathology of gonadal tumors [49,50]. In some cases, the discordance between our differential analysis (tumor vs. normal) and literature data may be due to the choice of GTEx normal samples to compare with each TCGA sample types. For instance, ocular melanoma showed a strong downregulation of IL6 expression levels comparing to normal tissue (GTEx normal skin) in contrast with literature [51,52]. However, it is noteworthy that our overall survival analysis revealed that endogenous IL6 expression levels were negatively correlated with patient survival in a significant number of tumors (n = 8), including uveal melanoma and lung cancers, indicating pro-tumoral role of IL-6, as reported in literature [53]. The release of IL-6 from tumor cells may modulate the activity of the inflammation and immunity cells in the tumor microenvironment [2]. Of note, high IL-6 expression is associated with poor prognosis in both non-Hodgkin’s and Hodgkin’s lymphoma [54,55]. Similarly, it was reported that IL-6 expression in epithelium and stroma tissues of primary CRC was related to tumor invasion depth [56].

In order to test the tumor responsiveness to IL6, the expression of IL6R isoforms and IL6ST were evaluated in all TCGA tumors. Interestingly, the expression of membrane-bound IL6R isoform was moderately upregulated in most tumor types although the total expression IL6R was largely downregulated. This result suggested that membrane isoform of IL6R is more expressed in some cancer types so sustaining the activation of IL-6 cis-signaling. However, the slight reduction of IL6ST observed in many tumors may partially affect the direct action of IL-6 on tumor cells. As a consequence, we can speculate that IL-6 also indirectly promotes tumor growth stimulating in paracrine fashion the tumor-surrounding cells to release pro-tumor mediators, including IL-6 [57].

Our bioinformatic analysis was also aimed to investigate if epigenetic control through DNA methylation and miRNAs may be involved in IL-6 pathways regulation in cancer. DNA methylation analysis revealed that only IL6R gene expression was affected by intragenic hypermethylation mapping at ~1500 bp after start codon in most TCGA tumors (Figure S5), suggesting a novel regulatory region near to the IL6R promoter. Along with the well-known mechanism of gene silencing by promoter methylation, the intragenic DNA methylation also modulates alternative splicing of several genes [24,25,26]. However, no significant evidences were observed analyzing the role of DNA methylation in alternative splicing of IL6R gene in TCGA cohort. Notably, we observed that the intragenic hypermethylation of IL6R was generally associated with a poor prognosis in different tumors, thus highlighting the specific role of IL6R expression in tumor progression. Our results on IL6R methylation deserve further validations studies to fill this current gap in the literature concerning cancer, IL6 signaling, and its epigenetic regulation.

In this analysis it emerged that IL6, IL6R, and IL6ST gene expression was also regulated by miRNAs. Despite the role of miRNAs in the IL6 pathway regulation having been investigated [5], our bioinformatics analysis revealed that the IL6 expression was negatively regulated by few miRNAs and in a restricted number of tumors, indicating a marginal role of this post transcriptional regulation in IL6 expression. Conversely, the IL6R and IL6ST expression was regulated by a large number of miRNAs with a similar trend in different tumor types, indicating that miRNAs epigenetically regulate mainly the IL6 receptor complex. Among the miRNAs with the highest influence on IL6 pathway, the miR-182-5p affected all three genes in different tumors. So far, many literature data reported the involvement of miR-182-5p in the tumor progression of liver, breast, ovarian, bladder, and colon cancer by modulating key cellular processes relevant for cancer, including angiogenesis, apoptosis, migration, and proliferation [58,59,60,61,62].

Furthermore, some of the miRNAs targeting IL6R and IL6ST were positively correlate with IL6, indicating that IL6 may repress the IL6 receptor complex expression as negative feedback on the IL6 signaling. Additionally, we identified 9 miRNAs targeting IL6R exon 9 that may be responsible for repressing translation of IL6R transmembrane isoform.

Targeting IL6/STAT3 axis represents a promising therapeutic strategy to counteract the tumor growth and invasion. The effectiveness of targeted biologic inhibitors of IL6 pathway mainly depends on the expression of IL6R as a membranous or soluble form which sustains the IL-6 trans-signaling. Several preclinical and clinical studies suggested that IL6R inhibitors, such as Tocilizumab, Sarilumab, and Olamkicept, can be effective in some cancer type including ovarian, pancreatic, breast cancer, and B cell chronic lymphocytic leukemia [7].

Based on these evidences, our results pave the way to identify new genetic and epigenetic biomarkers that are useful to predict the therapeutic response of tumor patients to biologic drugs direct towards IL6/STAT3 axis.

## 4. Materials and Methods

### 4.1. Sample and Datasets

The public TCGA Dataset was analyzed to obtain both the gene expression levels of IL6, IL6R, and IL6ST (IlluminaHiSeq pancan normalized) and the exon expression levels of IL6R (IlluminaHiSeq) in the main tumor types. In particular, the exon expression levels of IL6R exon 9 (chr1:154426964-154427057—GRCh37/hg19), containing the coding sequence of transmembrane domain, and exon 2 (chr1:154401672-154401920—GRCh37/hg19), included in all IL6R isoforms, were analyzed. Furthermore, the expression levels of all miRNAs (IlluminaHiseq) and DNA methylation (Methylation450k) data relative to IL6, IL6R, and IL6ST genes were retrieved from each TCGA tumor dataset. The relative position of CG methylation probesets within IL6, IL6R and IL6ST gene regions were shown in Figure S5.

To perform the differential gene expression analysis between tumors and normal tissues, the cohort TCGA TARGET GTEx containing cross-platform normalized expression levels (RNAseq RSEM norm-count) of both tumor (TCGA) and normal (GTEx) samples was used (Table 1). Cancer name abbreviations, sample size, and phenotypic characteristics of datasets included in this study are reported in Table 3. The TCGA cohorts COADREAD, FPPP, GBMLGG, and LUNG were excluded because of missing datasets. UCSC Xena tools (https://xena.ucsc.edu/, accessed on 12 January 2021) was used to retrieve genomic data and clinical pathological features of selected dataset [63].

### 4.2. Analysis of Gene and Exon Expression

The difference of IL6, IL6R, and IL6ST gene expression levels between the tumor and normal samples was reported as Fold Change:

$$\pm 2^{\left|\log2\left[\mathrm{Mean}\left(\mathrm{Tumor}\right)\right]-\log2\left[\mathrm{Mean}\left(\mathrm{Normal}\right)\right]\right|}.$$

The relative abundance of IL6R transmembrane isoform, expressed as FC, was calculated according to the formula:

$$\pm 2^{\left|\log2\left[\mathrm{Mean}\left(\mathrm{Exon}9\right)\right]-\log2\left[\mathrm{Mean}\left(\mathrm{Exon}\;2\right)\right]\right|}.$$

Statistical significance was calculated by T-test considering *p* ≤ 0.05 as significant.

In addition, the SpliceSeq tool (http://bioinformatics.mdanderson.org/TCGASpliceSeq/, accessed on 23 February 2021) was used to calculate the percent-spliced-in (PSI) value for splicing events of IL6R gene in all TCGA tumors [64]. The PSI values allowed to estimate the proportion of exon retention or skipping in each sample, e.g., a PSI value equal to 0.9 for IL6R Exon 9 indicates that 90% of IL6R transcripts including the Exon 9 (Figure S3).

### 4.3. Overall Survival and Progression Free Interval Analyses

For each TCGA tumor type, overall survival (OS) and progression free interval (PFI) analyses were performed considering the IL6, IL6R, and IL6ST gene expression, the methylation status of selected CG probesets and the expression of selected miRNAs, separately. Kaplan Meier Log-rank (Mantel-Cox) test was carried out using GraphPad (Version 8.0.2) (GraphPad Software, San Diego, CA, USA) and UCSC Xena portal dividing the tumor samples in two groups (up and down) on the median value [63]. The log rank test value allows to estimate the strength of the difference between survival curves for each comparison group [65]. Similarly, OS and PFI analyses were performed considering the median of relative expression of IL6R Exon 9 quantified as difference of Exon 9 and Exon 2 log2 values.

### 4.4. Correlation Analysis

Correlation analysis between IL6, IL6R, and IL6ST gene expression and either DNA methylation or miRNAs expression was performed by Pearson’s r correlation test. Similarly, DNA methylation and miRNA expression were correlated to Exon 9 and 2 expression to verify if Exon 9 skipping depends on epigenetic regulation in cancer. A *p*-value less than 0.05 was considered statistically significant. The open source Heatmapper tool (http://www.heatmapper.ca, accessed on 15 March 2021) was used to visualize correlation data across the TCGA tumor types [66].

### 4.5. miRNA Targets Analysis

The microRNA Data Integration Portal (mirDIP) (https://ophid.utoronto.ca/mirDIP, accessed on 17 March 2021) was used to identify the miRNAs that potentially target the IL6, IL6R, and IL6ST mRNAs [67]. Furthermore, the STarMirDB software (http://sfold.wadsworth.org/starmirDB.php, accessed on 18 March 2021) was chosen to predict the miRNAs targeting the IL6R Exon 9 sequences. The miRNAs targeting the CDS-seed and CDS-seedless sequences within IL6R Exon 9 were selected by LogitProb ≥ 0.5 [68].

## 5. Conclusions

Overall, our bioinformatic analysis identifies several epigenetic factors affecting IL6 signaling that may have a diagnostic, prognostic, and predictive value in inflammatory-related tumors. Furthermore, our findings on epigenetic mechanism involved in IL6R gene regulation allows us to predict the responsiveness to anti-IL6R inhibitors in several IL6-related diseases.

## Figures and Tables

**Table 1:** Gene expression of IL6, IL6R, and IL6ST and survival analysis in all tumor types.

Tumor Type	IL6			IL6R			IL6ST		
	T vs. N (FC)	OS *	PFI *	T vs. N (FC)	OS *	PFI *	T vs. N (FC)	OS *	PFI *
Acute Myeloid Leukemia (LAML)	−2.92		NA	−1.93		NA			NA
Adrenocortical Cancer (ACC)							−1.44		
Bile Duct Cancer (CHOL)			5.584 (+)						
Bladder Cancer (BLCA)	1.89			−3.51			−3.30		5.39 (−)
Breast Cancer (BRCA)	−7.37			−2.02					7.54 (+)
Cervical Cancer (CESC)	3.04			−2.61			−3.32		
Colon Cancer (COAD)				−5.70			−2.56		
Endometrioid Cancer (UCEC)	1.79			−3.30			−3.21		
Esophageal Cancer (ESCA)	1.62								
Glioblastoma (GBM)	2.66		5.89 (−)	1.40					
Head and Neck Cancer (HNSC)	2.20	4.57 (−)		1.79			−2.36	5.42 (+)	5.23 (+)
Kidney Chromophobe (KICH)	−2.44			−2.21					
Kidney Clear Cell Carcinoma (KIRC)	1.59	31.46 (−)	17.13 (−)	2.00	16.31 (+)	16.09 (+)	3.11	9.10 (+)	6.09 (+)
Kidney Papillary Cell Carcinoma (KIRP)		5.78 (−)	4.43 (−)						
Large B-cell Lymphoma (DLBC)	6.39			−10.49					
Liver Cancer (LIHC)	−1.58			−1.46					
Lower Grade Glioma (LGG)	−1.92	12.38 (−)	6.77 (−)		5.04 (−)		1.41		
Lung Adenocarcinoma (LUAD)	−4.08				6.50 (+)		−1.66	8.90 (+)	
Lung Squamous Cell Carcinoma (LUSC)	−2.40	4.87 (−)		−2.36			−3.60		
Melanoma (SKCM)				1.42					
Mesothelioma (MESO)	−2.80			−3.50	4.20 (+)	11.79 (+)			
Ocular melanomas (UVM)	−11.90	7.52 (−)	4.19 (−)	−1.46					
Ovarian Cancer (OV)	2.88						−2.54		
Pancreatic Cancer (PAAD)	3.21						1.42		
Pheochromocytoma and Paraganglioma (PCPG)				−3.97			−1.74		
Prostate Cancer (PRAD)				−1.87					
Rectal Cancer (READ)				−5.66			−2.43		
Sarcoma (SARC)		5.74 (+)		−15.19	6.92 (+)				
Stomach Cancer (STAD)	2.18	4.57 (−)		−1.56		4.59 (−)			4.16 (−)
Testicular Cancer (TGCT)	5.19			3.47					
Thymoma (THYM)				−11.77	3.87 (−)		2.69		
Thyroid Cancer (THCA)	−2.20	3.66 (−)		−1.59					
Uterine Carcinosarcoma (UCS)	1.52			−5.38			−2.89		

The Fold Change (FC) values ≥ 1.4 or ≤ −1.4 were showed in the table. OS and PFI analyses were performed considering the median of relative expression of each gene. The difference between the survival distributions of two expression groups was expressed as Chi-square values (Mantel-Cox test). All results were statistically significant (*p* ≤ 0.05). Abbreviations: T: Tumor sample; N: Normal sample; FC: Fold Change; OS: Overall Survival; PFI: Progression Free Interval; *: Chi-square values; NA: Not applicable; (+): favorable; (−): unfavorable.

**Table 2:** Survival analysis of TCGA patients based on methylation status of selected IL6 and IL6R CG probesets.

Cancer Type	IL6				IL6R					
	cg13104385		cg05265849		cg09257526		cg04437762		cg05756780	
	OS *	PFI *	OS *	PFI *	OS *	PFI *	OS *	PFI *	OS *	PFI *
Acute Myeloid Leukemia (LAML)	5.42 (+)									
Adrenocortical Cancer (ACC)					21.70 (−)	18.51 (−)	8.68 (−)	18.77 (−)	4.12 (−)	
Bile Duct Cancer (CHOL)										6.66 (−)
Bladder Cancer (BLCA)										4.72 (−)
Breast Cancer (BRCA)					5.31 (+)					
Cervical Cancer (CESC)							4.25 (−)	5.53 (−)	5.28 (−)	5.90 (−)
Endometrioid Cancer (UCEC)		7.10 (+)							5.39 (−)	4.90 (−)
Glioblastoma (GBM)								4.58 (+)		
Head and Neck Cancer (HNSC)										4.60 (−)
Kidney Clear Cell Carcinoma (KIRC)						4.93 (−)				4.09 (−)
Kidney Papillary Cell Carcinoma (KIRP)	4.34 (+)	5.44 (+)					10.41 (−)	6.56 (−)	7.25 (−)	
Lower Grade Glioma (LGG)	21.47 (+)	17.17 (+)	8.01 (+)	11.80 (+)	22.81 (+)	19.12 (+)	5.53 (+)			
Lung Adenocarcinoma (LUAD)					5.18 (−)	6.92 (−)	4.03 (−)		13.40 (−)	7.39 (−)
Lung Squamous Cell Carcinoma (LUSC)	4.37 (+)	11.68 (+)		4.17 (+)						
Melanoma (SKCM)	5.75 (−)					5.58 (+)			10.46 (+)	4.74 (+)
Mesothelioma (MESO)					5.00 (−)	9.43 (−)	14.36 (−)	27.43 (−)		9.02 (−)
Ocular melanomas (UVM)							4.28 (−)	7.39 (−)	18.55 (−)	14.88 (−)
Pancreatic Cancer (PAAD)								4.29 (−)		
Sarcoma (SARC)		4.15 (+)							5.06 (−)	
Thymoma (THYM)									12.19 (+)	

OS and PFI analyses were performed considering the methylation median levels of selected CG probesets. The difference between the survival distributions of two methylation groups was expressed as Chi-square values (Mantel-Cox test). In the table only tumors showing at least one statistically significant value were included (*p* ≤ 0.05). Abbreviations: OS: Overall Survival; PFI: Progression Free Interval; *: Chi-square values; (+): favorable; (−): unfavorable.

**Table 3:** TCGA and GTEx datasets and number of samples available in each dataset analyzed in this study.

Tumor Type (Abbreviation)	TCGA/GTEx		TCGA †				
	Tumor TCGA (N)	Normal GTEx (N)	Total (N)	Gene expr. (N)	DNA Methylation (N)	miRNA expr. (N)	EXON expr. (N)
Acute Myeloid Leukemia (LAML)	173	Blood (337)	200	173	194	188*	173
Adrenocortical Cancer (ACC)	77	Adrenal Gland (128)	92	79	80	79	79
Bile Duct Cancer (CHOL)	36	Liver (110)	45	45	45	45	45
Bladder Cancer (BLCA)	407	Bladder (9)	436	426	434	429	426
Breast Cancer (BRCA)	1099	Breast (179)	1247	1218	888	832	1218
Cervical Cancer (CESC)	306	Cervix Uteri (10)	313	308	312	311	308
Colon Cancer (COAD)	290	Colon (308)	551	329	337	261	329
Endometrioid Cancer (UCEC)	181	Uterus (78)	596	201	478	430	201
Esophageal Cancer (ESCA)	182	Esophagus (653)	204	196	202	195	196
Glioblastoma (GBM)	166	Brain (1141)	629	172	155	5	172
Head and Neck Cancer (HNSC)	520	Salivary Gland (55)	604	566	580	529	566
Kidney Chromophobe (KICH)	197	Kidney (29)	91	91	66	89	91
Kidney Clear Cell Carcinoma (KIRC)	531	Kidney (29)	945	606	480	311	606
Kidney Papillary Cell Carcinoma (KIRP)	289	Kidney (29)	352	323	321	321	323
Large B-cell Lymphoma (DLBC)	47	Blood (337)	48	48	48	47	48
Liver Cancer (LIHC)	371	Liver (110)	438	423	429	420	423
Lower Grade Glioma (LGG)	523	Brain (1141)	530	530	530	524	530
Lung Adenocarcinoma (LUAD)	515	Lung (288)	706	576	492	495	576
Lung Squamous Cell Carcinoma (LUSC)	498	Lung (288)	626	553	415	380	553
Melanoma (SKCM)	469	Skin (556)	481	474	476	452	474
Mesothelioma (MESO)	87	Lung (288)	87	87	87	87	87
Ocular melanomas (UVM)	79	Skin (556)	80	80	80	80	80
Ovarian Cancer (OV)	427	Ovary (88)	630	308	10	485	308
Pancreatic Cancer (PAAD)	179	Pancreas (167)	196	183	195	182	183
Pheochromocytoma & Paraganglioma (PCPG)	182	Adrenal Gland (128)	187	187	187	186	187
Prostate Cancer (PRAD)	496	Prostate (100)	566	550	549	544	550
Rectal Cancer (READ)	93	Colon (308)	186	105	106	92	105
Sarcoma (SARC)	262	Muscle (396)	271	265	269	260	265
Stomach Cancer (STAD)	414	Stomach (174)	580	450	398	428	450
Testicular Cancer (TGCT)	154	Testis (165)	156	156	156	155	156
Thymoma (THYM)	119	Blood (337)	126	122	126	126	122
Thyroid Cancer (THCA)	512	Thyroid (279)	580	572	571	569	572
Uterine Carcinosarcoma (UCS)	57	Uterus (78)	57	57	57	56	57

†: Including both tumor and normal samples, *: IlluminaGA dataset.

## Data Availability

Not applicable.

## Supplementary Materials

The following are available online at https://www.mdpi.com/article/10.3390/ijms221810172/s1.
