# Supplementary material for: Novel Insights into Epigenetic Regulation of IL6 Pathway: In Silico Perspective on Inflammation and Cancer Relationship
Source: Int J Mol Sci. 2021 Sep 21;22(18):10172. doi: 10.3390/ijms221810172 (PMC8470126; doi:10.3390/ijms221810172)
Supplement: Supplementary file 1 [file ijms-22-10172-s001.zip › Supplementary figures IJMS.pdf]

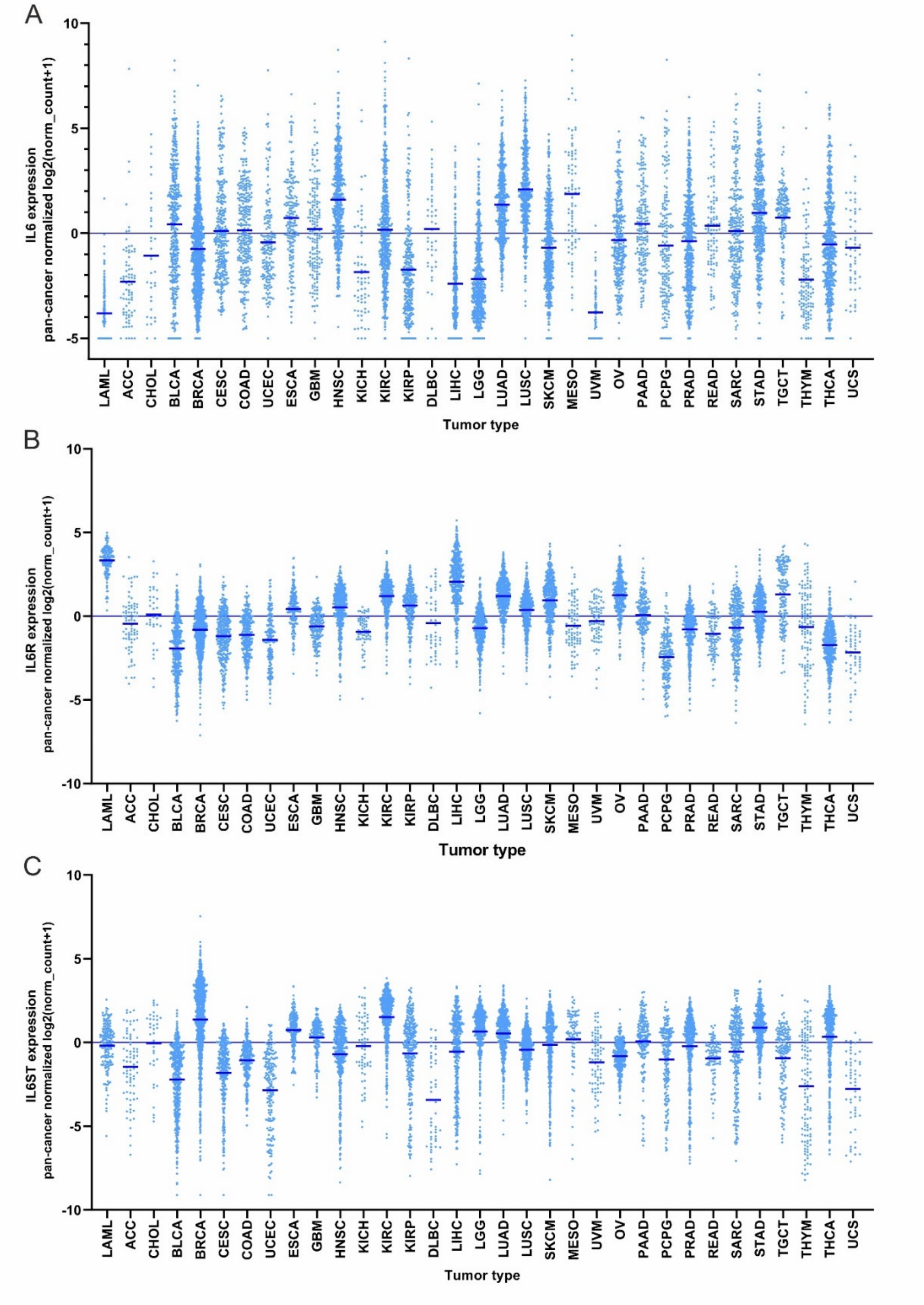

Figure S1. Expression levels of IL6 (A), IL6R (B) and IL6ST (C) for each TCGA cohort. The expression data were retrieved from UCSC Xena tool.

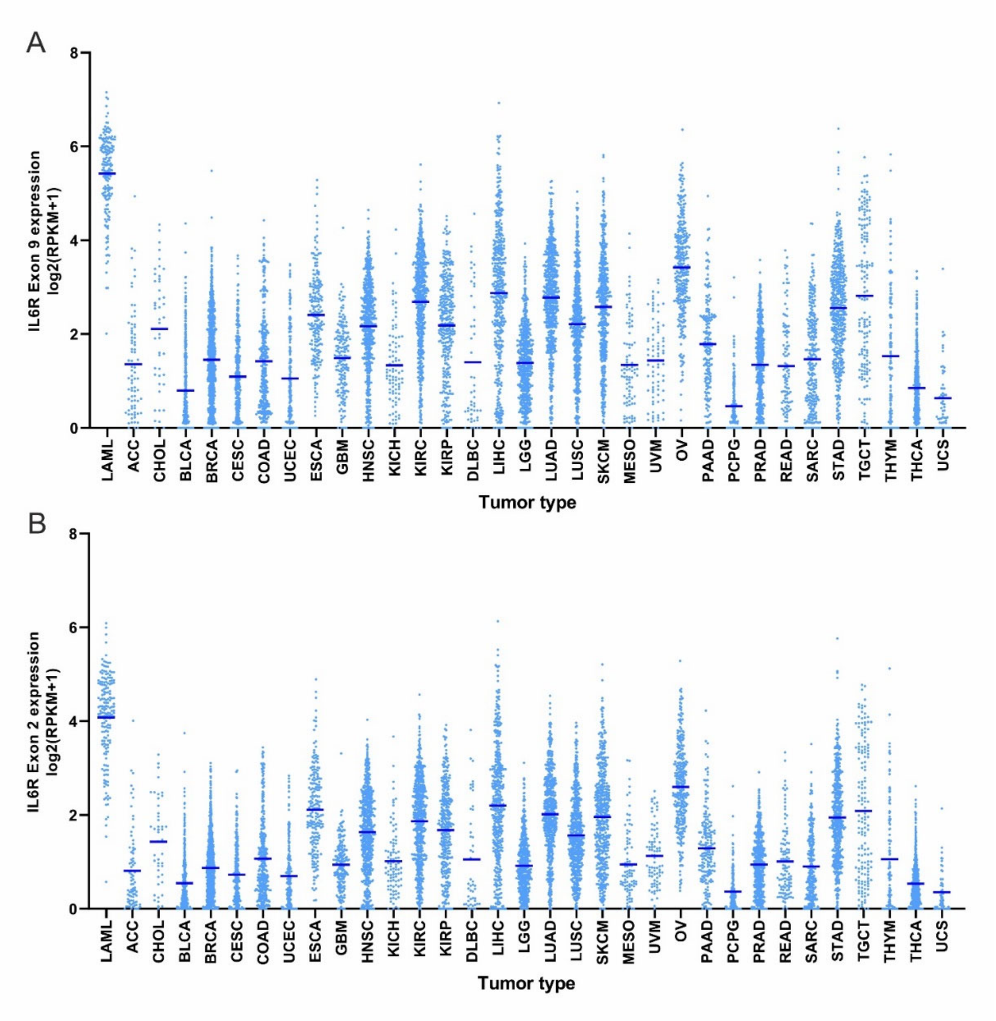

Figure S2. Exon expression levels IL6R Exon 9 (A) and IL6R Exon 2 (B) for each TCGA cohort. The Exon expression data were retrieved from UCSC Xena tool.

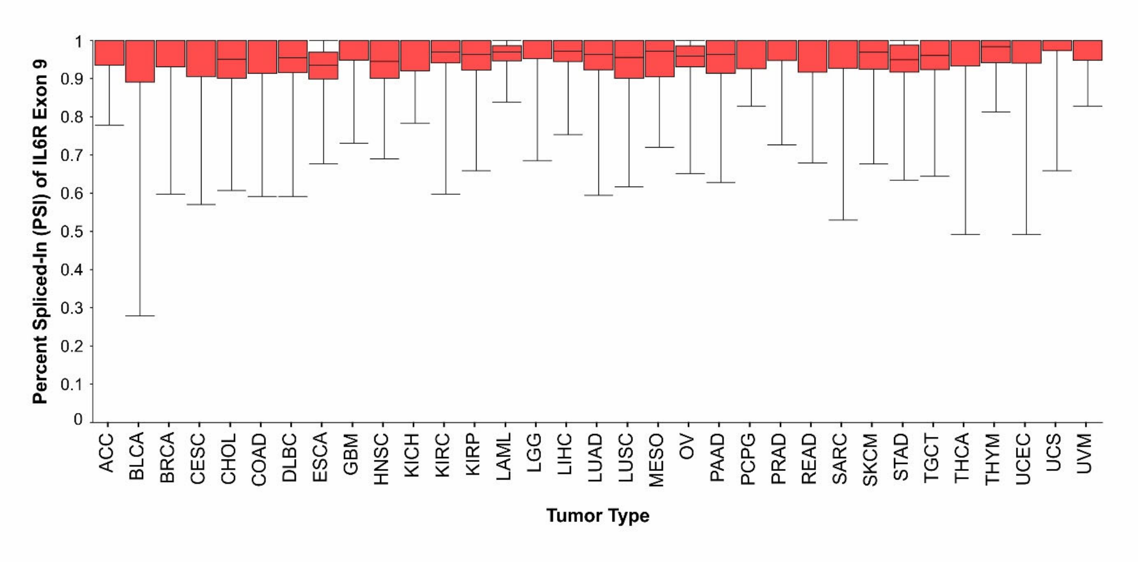

Figure S3. SpliceSeq analysis of IL6R gene. The percent-spliced-in (PSI) value for splicing events of IL6R Exon 9 were evaluated in all TCGA tumors.

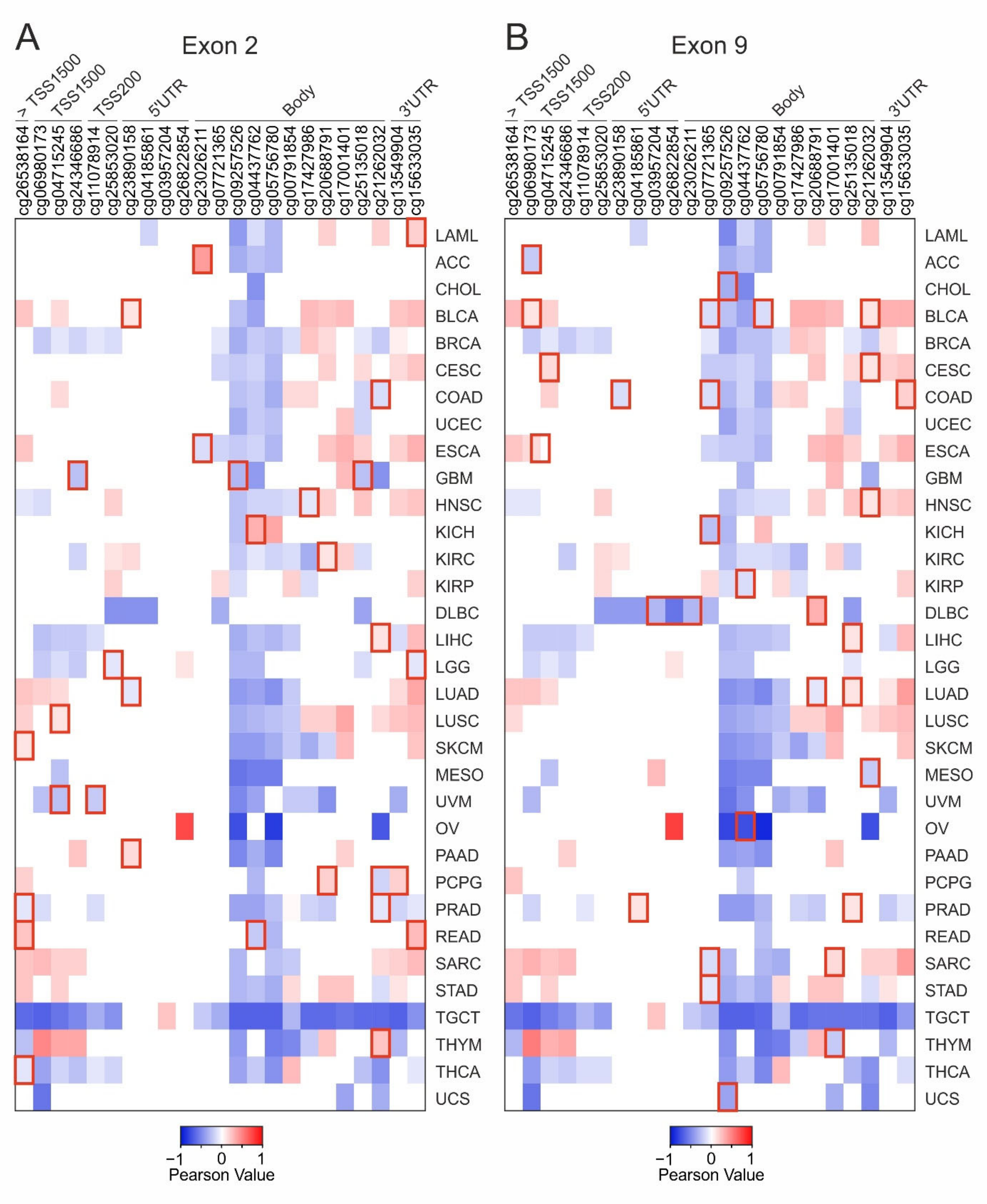

Figure S4. Correlation analysis between IL6R Exon 2 and 9 expression and DNA methylation for each tumor type. The heatmap shows the correlation (Pearson's $r \leq -0.3$ or ≥ 0.3, $p \leq 0.05$) between and methylation levels of CG probesets relative to IL6R gene and Exon 9 (A) and Exon 2 (B) expression, separately. The red outlines highlight the CG probesets correlation values that are discordant or mutually exclusive among Exon 2 (A) and Exon 9 (B) correlation analysis.

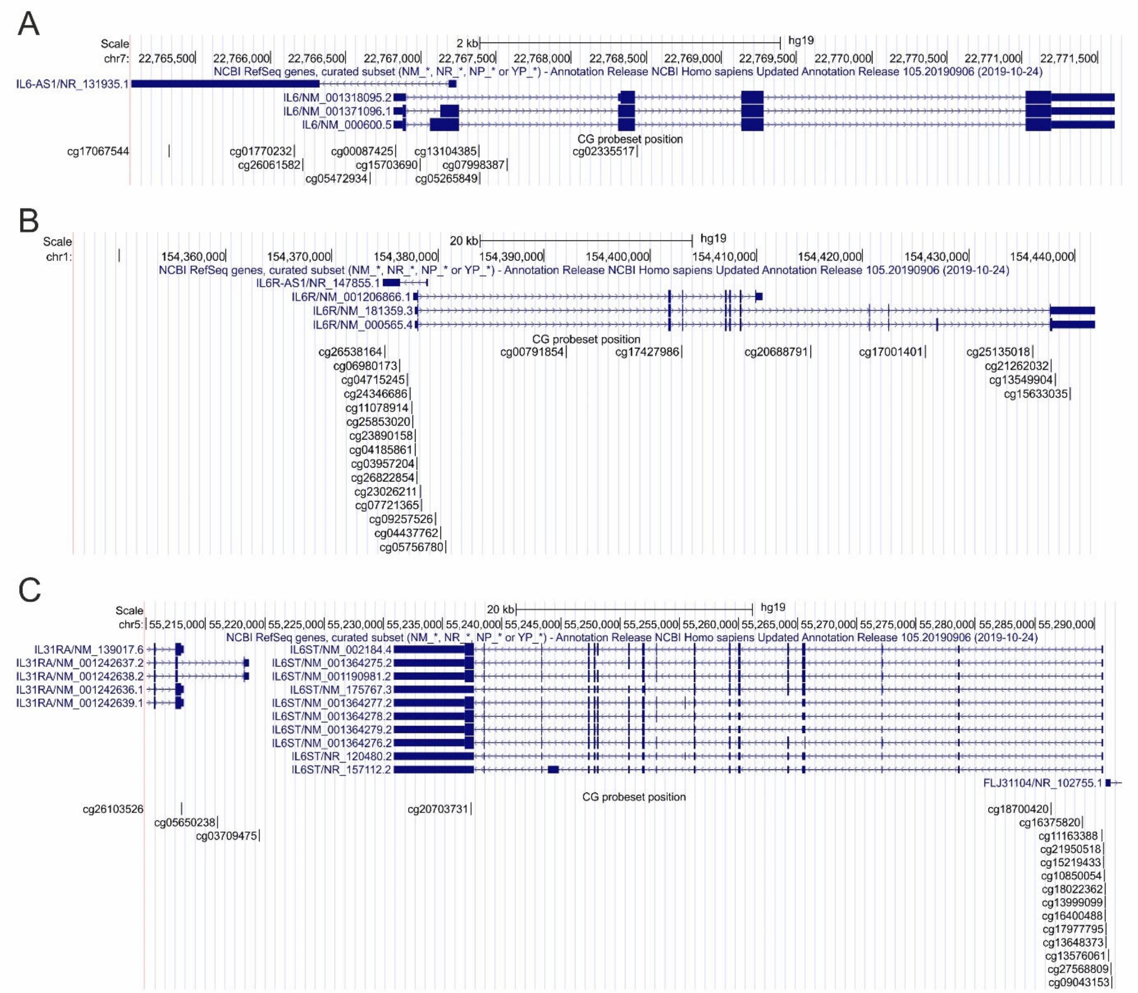

Figure S5. Genome position of CG probesets of IL6, IL6R and IL6ST. UCSC portal (Assembly: GRCh37/hg19) was used to retrieve the genomic position of CG probeset within IL6 (A) IL6R (B) and IL6ST (C) loci. RefSeq gene annotation (NCBI: Annotation Release 105.20190906) was used to display the isoforms of each gene.
